# Calcific myofibrosis due to pentazocine abuse: a case report

**DOI:** 10.1186/1752-1947-2-160

**Published:** 2008-05-17

**Authors:** Vinay Goyal, Jatinder M Chawla, Yatan PS Balhara, Garima Shukla, Sumit Singh, Madhuri Behari

**Affiliations:** 1 Department of Neurology, All India Institute of Medical Sciences, New Delhi 110029, India; 2 Department of Psychiatry and National Drug Dependence Treatment Center, All India Institute of Medical Sciences, New Delhi 110029, India

## Abstract

**Introduction:**

Pentazocine, a synthetic narcotic analgesic, is commonly used for the relief of moderate to severe pain secondary to various conditions. It is usually well tolerated; however, adverse effects are not uncommon, especially when higher doses are used and when it is used in a dependent fashion. There have been reports of various complications associated with its use, including skin fibrosis, skin ulceration, abnormal skin pigmentation and symmetrical myopathy with fibrous myopathy. Fibrosis has usually been reported in the muscles at the site of injection of the drug. Being opioid in nature, it has a high abuse potential.

**Case presentation:**

Here we report a case of pentazocine-induced calcific myofibrosis in a 42-year-old man involving muscles which were not injected with pentazocine.

**Conclusion:**

This case highlights the care that needs to be taken when prescribing opioid analgesics, such as pentazocine, as routine painkillers. Patients who have history of substance abuse are more likely to abuse other agents, including prescription drugs. Rare consequences such as calcific myofibrosis are devastating and can cause significant lifelong disability.

## Introduction

Pentazocine is a synthetic narcotic analgesic used chiefly for the relief of moderate to severe pain. There have been reports of various complications associated with its use, including skin fibrosis, skin ulceration, abnormal skin pigmentation and symmetrical myopathy [1,2] with fibrous myopathy (a rare complication following prolonged pentazocine injection) [3,4]. Fibrosis has usually been reported in the muscles at the site of injection of the drug. The association of myopathy with contractures around the shoulder and hip joints is rare [5,6]. Here we report a case of pentazocine-induced calcific myofibrosis involving muscles mainly around the hip, shoulder, elbow and knee joints following long-standing pentazocine use in a dependent fashion. In this particular case, muscles which were not used for injection of pentazocine have shown pathological changes.

## Case presentation

We present the case of a 42-year-old right-handed man, admitted with complaints of painless and progressive persistent stiffness along with wasting of the muscles of the back and proximal limbs for the previous 6 years. There was significant impairment of his daily activities including walking, bending (forward, backward and sideways), lifting of arms and so on. The impairment was to the extent that he needed assistance in rising from the supine position. Due to the involvement of pelvic girdle muscles, his gait had become short-stepping. Also, he could not abduct his thighs more than 20°, contributing to significant gait disability. There was no associated weakness.

Six years previously, the patient was given a pentazocine injection by a local physician for abdominal pain with a presumed diagnosis of pancreatitis. It helped the patient and he subsequently used pentazocine intramuscularly in a dependent fashion for 6 years. The patient took pentazocine in combination with phenergan injection, around two ampoules (60 mg pentazocine) every day in divided doses. He injected the combination into muscles, preferentially into the buttocks and upper arms. Over this time, the patient never injected into thighs, calves, abdominal, shoulder girdle or forearm muscles. The injections were discontinued 6 months after the onset of above-mentioned symptoms. Previously, the patient had also consumed alcohol in a dependent fashion for 13 years, but stopped after experiencing abdominal pain. He was normotensive and non-diabetic.

**Figure 1:**
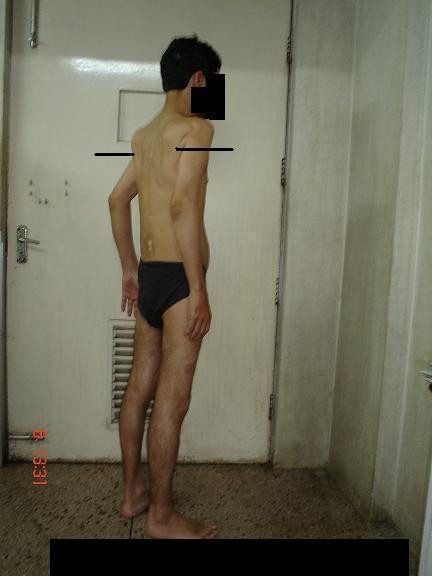
Wasting of the muscles around the shoulder joint.

**Figure 2:**
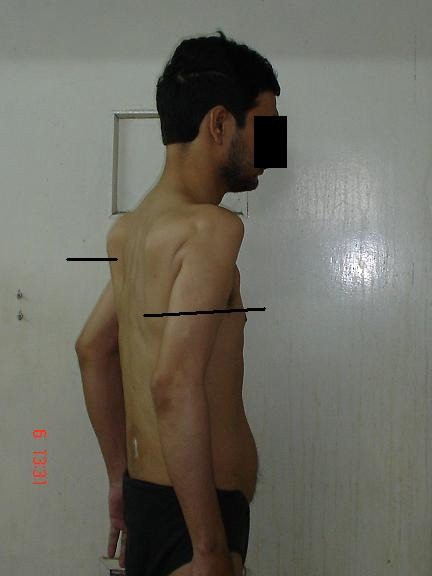
Wasting of the muscles around the shoulder joint (magnified view).

Physical examination of the patient revealed that he had great difficulty in rising from the supine position and bending from a standing position. There was wasting and hardening of the muscles of the back, proximal arms and thighs (Figures 1, 2). He walked with a lordotic gait and had marked woody indurations of the deltoids, biceps, glutei and quadriceps. The range of movements was decreased markedly. His arms could not be actively abducted beyond 45° to 50° and the legs not more than 20°. Both elbows were semi-flexed with no more than a 15° range of movement. He was unable to cross his legs and was not able to touch his back with his hands. Active thigh flexion was limited to 10°. Movements at the distal joints in both upper and lower limbs were normal. Muscle power was normal within the limited range of movements, and there was no sensory deficit. Examination of the rest of the nervous and other systems did not reveal any other abnormalities.

**Figure 3:**
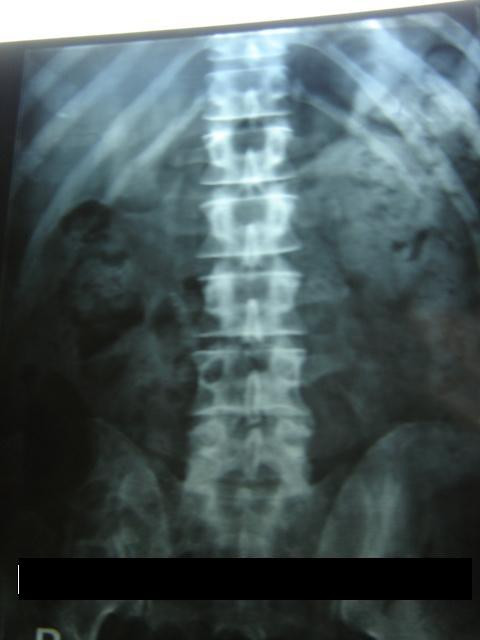
X-Ray of the paraspinal and pelvic region showing calcification.

**Figure 4:**
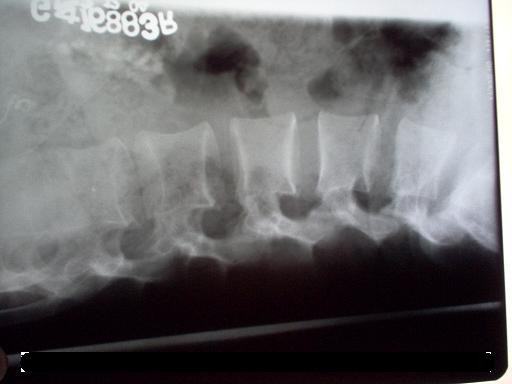
X-Ray showing calcification in the paraspinal region.

On investigation, a full blood count, liver and renal function tests, serum calcium, phosphate, and creatinine phosphokinase were within the normal ranges. Roentgenogram of the lumbar-sacral spine, thigh, knee, chest, shoulder and cervical spine showed multiple soft tissue calcifications with hyperdensity of muscles (Figures 3, 4). There was no articular abnormality. Electromyographic examination of muscles was normal. Muscle biopsy showed atrophy with features suggestive of neurogenic involvement without active inflammatory signs.

## Discussion

Clinical presentation of the case produced various differential diagnoses including ankylosing spondylitis, Stiff-man syndrome, myositis ossificans and parathyroid disease. Ankylosing spondylitis was ruled out as there was no involvement of the joints. Stiff-man syndrome presents with spasms and cramps, and usual presentation is after middle age. These features ruled out the possibility of this syndrome. The possibility of myositis ossificans was unlikely, as the present case was of late onset and was characterized by the absence of skeletal abnormality. Normal serum calcium and phosphate levels excluded hypoparathyroidism. Pentazocine-induced calcific myofibrosis was a strong possibility in view of the history of pentazocine abuse, calcified muscles and the clinical presentation.

Schlicher et al. and Swanson et al. first described the cutaneous complications of pentazocine injections and noted a 33% incidence of browny induration of skin and underlying tissues [1,7]. Steiner et al. and Joong et al. described fibrous myopathy with intramuscular pentazocine injections [3,4], with their patients presenting with woody induration of muscles with secondary contractures.

The exact mechanism of this condition remains elusive. Pentazocine is acidic (pH 4.3) in nature and its crystals precipitate easily in a neutral or slightly alkaline medium. This property, along with the muscle trauma caused by repeated needling and rapid injections of large boluses of drugs, may be responsible for this or other types of drug-induced myopathy [8]. This remains the most explicable and acceptable explanation of the condition.

The use of pentazocine in this case was associated with the use of phenergan injection. This could have played a contributory role to the phenomenon, although we do not consider it as the primary causative agent since its use has not been associated with such lesions in the literature, whereas pentazocine has been associated with such phenomena in the directly injected muscles.

Another interesting aspect of this case, which has not been frequently observed, is the involvement of muscle groups which were not injected with pentazocine. The patient's self-reported history and the inaccessibility of the muscle groups involved (those of the shoulder blade) support the claim that these muscle groups were not directly injected by the patient. The local action of pentazocine, as proposed, seems to be an unlikely explanation of the condition. The presence of fibrosis and calcification in distant muscles rather suggests a different mechanism. The possible mechanism could be a direct action of the drug once it enters into the circulation or the release of an independent factor from the site of injection that leads to widespread involvement of the muscles.

Moreover, the onset of the fibrotic changes correlated to the use of the injection pentazocine and was temporally unrelated to the past use of the alcohol, as the patient and his family members corroborated that he had stopped alcohol use at least 6 years previously. Moreover, we could find no complications caused by long-term use of alcohol, and the patient's full blood count revealed normal red cell indices which supported abstinence from alcohol in recent times.

Prescription drug abuse is a major health problem across the globe. Various drugs, such as analgesics, cough syrups, vitamin preparations and laxatives among others, are being used by individuals for reasons other than the medical indication. The abuse of prescription opioids, such as pentazocine, is being increasingly reported across globe [9] including India [8,10,11]. The availability of these drugs over the counter precludes the requirement of a prescription to procure them. With free over-the-counter access to these drugs in India and many developing countries, awareness of this complication is important so that unwanted side effects can be avoided. Moreover, in cases such as that reported here, the drugs are initially prescribed for a medical indication and subsequent use by the patient continues without the advice of a physician. Clinicians should be vigilant about the possibility of these compounds being used in this way, and extra caution should be exercised when dealing with individuals with a history of substance abuse and/or dependence. This will help in preventing such drug abuse and its complications.

## Conclusion

This case highlights the significance of the care that should be taken when prescribing opioid analgesics, such as pentazocine, as routine painkillers. Patients who have history of substance abuse are more likely to abuse other agents, including prescription drugs. Rare consequences such as calcific myofibrosis are devastating and can cause significant lifelong disability.

## Competing interests

The authors declare that they have no competing interests.

## Authors' contributions

All the authors have made significant contributions to the manuscript as per the journal guidelines. All the authors have read and approved the final manuscript.

## Consent

Written informed consent was obtained from the patient for publication of this case report and accompanying images. A copy of the written consent is available for review by the Editor-in-Chief of this journal.
